# Phytochemical and Antinociceptive, Anti-Inflammatory, and Antioxidant Studies of* Smilax larvata* (Smilacaceae)

**DOI:** 10.1155/2016/9894610

**Published:** 2016-12-22

**Authors:** Beatriz Cristina Konopatzki Hirota, Cristiane da Silva Paula, Vinícius Bednarczuk de Oliveira, Joice Maria da Cunha, Anne Karoline Schreiber, Fernanda Maria Marins Ocampos, Anderson Barison, Obdulio Gomes Miguel, Marilis Dallarmi Miguel

**Affiliations:** ^1^Pharmacotechnical Laboratory, Department of Pharmacy, Universidade Federal do Paraná, 80.210-170 Curitiba, PR, Brazil; ^2^Phytochemistry Laboratory, Department of Pharmacy, Universidade Federal do Paraná, 80.210-170 Curitiba, PR, Brazil; ^3^Laboratory of Pharmacology of Pain, Department of Pharmacology, Universidade Federal do Paraná, 80.210-170 Curitiba, PR, Brazil; ^4^NMR Center, Chemistry Department, Universidade Federal do Paraná, 80.210-170 Curitiba, PR, Brazil

## Abstract

The tea of aerial parts of* Smilax larvata* Griseb. (Smilacaceae) has been ethnopharmacologically used in Southern Brazil due to its anti-inflammatory action. In this study, ethanolic and organic extracts from aerial parts of* S. larvata* were phytochemically and pharmacologically characterized. The phytochemical analysis of EtOAc extract of* S. larvata* revealed the presence of three flavonoids, drabanemoroside, kaempferol 3-*O*-*α*-L-rhamnopyranosyl(1→2)-*α*-L-rhamnopyranoside, and kaempferol, the first two being isolated for the first time in this genus, two phenolic compounds* p-*hydroxybenzoic acid and* p-*coumaric acid, and alkaloids. In vitro assays demonstrated a potential antioxidant property of SLG. The treatment with SLG induced a significant reduction of the formalin-evoked flinches in rats, an effect reversed by opioid antagonist naloxone. Treatment with SLG also induced a significant increase in the hot plate latency and a decrease of intestinal motility by 45%. No effect was observed over nociceptive responses induced by a TRPA1 agonist mustard oil or over acetic acid-induced writhing in mice. Together, our data suggested that SLG has an in vivo antinociceptive effect, which seems to be associated with the opioid system activation. These findings support previous claims of medical use of* Smilax larvata* in the treatment of pain conditions.

## 1. Introduction

The genus* Smilax* belongs to the Smilacaceae family and comprises about 350 species of herbaceous plants, climbing and vines, also described as shrubs with creeping high branches [1, 2]. Distributed in tropical and subtropical regions,* Smilax* species are extensively used in traditional medicine around the world and in Brazil for the treatment of rheumatism and syphilis and as a diuretic [3, 4].

Previous investigations have already pointed out several biological activities of* Smilax* species extracts, such as anti-inflammatory, antinociceptive, antifungal, estrogenic, antiestrogenic, diuretic, and antihyperuricemic properties [5–8]. In this way, compounds with anti-inflammatory as well as antioxidant and antiarthritic activities were isolated from this genus, among them Smilaxchinoside A–D, Sieboldogenin, and Astilbin [9–11]. Phytochemical studies revealed the presence of saponins, flavonoids, phenolic compounds, tannins, alkaloids, and phenylpropanoids glucosides [2, 6, 7, 12].

Important for this study,* Smilax larvata* Griseb. is a climbing plant endemic from Brazil [13], popularly known as “cat's claw,” due to the presence of thorns in the steams, or as “salsaparrilha.” The tea of its aerial parts is ethnopharmacologically used as anti-inflammatory agent in Southern Brazil. Despite the traditional use, the pharmacological validation about* S. larvata* potential anti-inflammatory/antinociceptive activity has not been reported yet. A preliminary investigation about the potential biological activities of the* S. larvata* extracts reported the antifungal and larvicide activities of its crude ethanolic extract [14]. In the continuous search for active biological compounds, this work for the first time investigated furtherly potential anti-inflammatory, antinociceptive, and antioxidant properties as well as the chemical composition of* S. larvata*.

## 2. Material and Methods

### 2.1. General Procedures

Silica gel 60 (70–230 mesh) from Merck® was used for column chromatography. Preparative high performance liquid chromatography separations were performed on a Merck Elite LaChrom chromatograph composed of a quaternary solvent delivery system (model L-2130), a UV-DAD detector (model L-2455), and a loading valve fitted with a 100 *μ*L sample loop. The chromatograph was equipped with RP_18_ reversed-phase column (7.8 mm i.d. × 30 cm long and 10 *μ*m particle diameter) with a small precolumn (7.8 mm i.d. × 5 cm long) containing the same packing. NMR experiments were acquired on either Bruker AVANCE III HD 600 or AVANCE III 400 or a DPX 200 NMR spectrometer operating at 14.1, 9.4, and 4.7 T, observing ^1^H and ^13^C at 600.13, 400.13, and 200.13 and 150.90, 100.61, and 50.32 MHz, respectively. The Bruker AVANCE III HD 600 spectrometer was equipped with 5 mm quadrinuclear inverse detection probe with *z* gradient. The Bruker AVANCE III 400 spectrometer was equipped with either a 5 mm multinuclear inverse detection probe with *z* gradient or a 5 mm multinuclear direct detection probe with *z* gradient. The DPX 200 instrument was equipped with a 5 mm quadrinuclear direct detection probe. The ^1^H and ^13^C {^1^H} spectra were acquired with spectra of ~11 ppm and ~240 ppm, respectively. One-bond (HSQC) and long-range (HMBC) ^1^H-^13^C NMR correlation experiments were optimized for average coupling constants ^1^ *J*_(H,C)_ and ^LR^ *J*_(H,C)_ of 140 and 8 Hz, respectively. All ^1^H and ^13^C NMR chemical shifts are given in ppm related to TMS signal at 0.00 ppm as internal reference. All chemicals were of analytical grade, while for HPLC methanol was of spectroscopy grade and water was obtained from an ultrapurification system. In biological assays, UV measurements were performed on a Shimadzu UV-1601 spectrophotometer.

### 2.2. Botanical Material

Aerial parts (leaves, flowers, and stems) of* Smilax larvata* were collected in November 2010 and 2011 in Curitiba, Paraná, Brazil (coordinates 25°26′45′′S and 49°20′51′′W), and identified by Osmar dos Santos Ribas, curator of The Botanical Museum of Curitiba, where a voucher specimen was deposited (#386302).

### 2.3. Extraction and Isolation

Dried and powdered aerial parts (80 g) were macerated with EtOH and submitted to a qualitative phytochemical screening [15], while 940 g was extracted with EtOH-H_2_O (90 : 10 v/v) with the aid of a Soxhlet system. The resulting extract was filtered (80 g/m^2^) and the solvent was removed under reduced pressure, giving the crude EtOH extract of* Smilax larvata* Griseb. (SLG) (145 g). Part of the SLG (140 g) was submitted to liquid-liquid extraction, giving* n*-hexane (15 g), CHCl_3_ (3.7 g), EtOAc (3.4 g), and EtOH-H_2_O (50.5 g) extracts. The EtOAc extract (2.4 g) was subjected to column chromatography eluted with a gradient system consisting of increasing concentration of EtOAc in* n*-Hex (50% v/v), followed by MeOH in EtOAc (until 100% MeOH), affording 150 fractions. Fraction 3 showed yellow crystals (15 mg) which were submitted to NMR analysis, giving a mixture of compounds** 3**,** 4,** and** 5**. Fraction 72 was submitted to a preparative HPLC purification eluted with MeOH-H_2_O at a flow rate of 3 mL/mL as follows: 40% of methanol in the initial 15 min followed by a gradient to 45% of methanol until 16 min and kept in this proportion until 30. Then, the proportion of methanol was raised to 95% in 10 min and kept for additional 11 min. After that, initial condition was reestablished in 2 min, resulting in compounds** 1** (34.4 mg) and** 2 **(18.5 mg).

### 2.4. Animals

Adult male Wistar rats (180–220 g) or Swiss albino mice (18–35 g), provided by Federal University of Paraná colony, were used in the present study. Animals were housed in temperature-controlled room (22 ± 2°C) under a 12/12 h light/dark cycle (lights on at 7 am) in a sawdust-lined plastic cage with free access to standard laboratory chow and tap water. All behavioral experiments were conducted during the light phase of the cycle (between 9 am and 4 pm) at least one hour after habituation to the experimental room. In days of experiments, animals were maintained in food deprivation for 12 hours and then, different doses SLG was administered by oral gavage. The study was conducted in accordance with the National Institutes of Health Guide for the Care and Use of Laboratory Animals (NIH Publication #8023, revised 1978) and approved by the local Ethics Animal Use Committee (CEUA/BIO-UFPR; #704). All efforts were made to minimize the number of animals and their suffering. Accordingly, following the reduction principle recommended by Russell and Burch (1959) [16], we did not conduct positive control groups during the behavioral experiments since the effect of drugs such as NSAIDs, steroidal drugs, or morphine has been extensively observed in previous studies [17].

### 2.5. Anti-Inflammatory Assay (Carrageenan-Induced Paw Edema)

The potential effect of the SLG extract on acute inflammation was evaluated in carrageenan-induced paw edema model, according to the method described previously by Pereira et al., 2012 [18]. After a 12-hour fasting period, four groups of six to eight rats each were orally treated with SLG (100, 200, or 400 mg/Kg) or vehicle (1% Teen 80, equivalent volume). These doses were chosen based on previous in vivo studies using extracts from other species of* Smilax* [5, 19]. One hour after the SLG or vehicle treatment, edema was induced by intraplantar injection of carrageenan (Cg; 200 *μ*g/paw; in 0.1 mL of saline). The contralateral paws were used as controls and received an equivalent volume of saline (0.1 mL). The paw thickness was measured before (basal measurement) and 1, 2, 3, 4, and 5 h after Cg or saline injection using a caliper rule. The edema is reported as the increasing in the paw thickness, calculated by subtracting the paw thickness measured at each time point after Cg or saline injection to the initial paw thickness (basal). The data were also expressed as area under curves using the trapezoid method (AUC, in arbitrary units).

### 2.6. Antinociceptive Assays

#### 2.6.1. Formalin Test

To evaluate the potential antinociceptive effect of the SLG, formalin test was performed according to Hunskaar and Hole (1987) [20]. Briefly, rats (*n* = 6–8 per group) submitted for a 12-hour fasting period were acclimated for at least 15 min to the testing environment by placing them in the formalin test apparatus (an inverted 280 mm wide and 400 mm high glass funnel) with a mirror positioned at an angle of 45° to allow unhindered observation of the animals' paws. Then, animals were orally treated with vehicle (1% Teen 80, 5 mL/Kg) or SLG (100, 200, or 400 mg/Kg) and, one hour later, each animal received a single formalin injection (2.5%, 50 *μ*L/paw) into the dorsum of one hind paw. Defined flinches were scored immediately after the formalin injection and continued for the next 60 min, divided into periods of 5 min with the phases defined as the following time bins: phase I, 1–10 min after injection, and phase II, 15–60 min after injection. Comparisons of behavior during each phase were made by summing the flinches recorded at measurement points within the phase.

#### 2.6.2. Acetic Acid-Induced Writhing

Since the acetic acid-induced writhing test has been extensively used for screening potential analgesic activities, the effect of the SLG was also evaluated in this test according to the method previously described by Shu et al. (2006) [3]. Briefly, male mice were divided into four experimental groups (*n* = 6–8/group): vehicle (1% Teen 80, 5 mL/Kg;* p.o.*) or SLG in three different doses (164, 327, and 654 mg/Kg;* p.o.*), calculated based on basal metabolic rate using the general method of calculation for interspecific allometric scaling of drug doses [21]. One hour later, the animals received an intraperitoneal injection of acetic acid (0.6%; 10 mL/Kg) and the number of writhings was counted cumulatively for 30 minutes.

#### 2.6.3. Hot Plate

To evaluate a possible central analgesic effect of SLG, rats (*n* = 6–8 per group) were placed on the heated surface of the hot plate apparatus (50 ± 1°C) before and one hour after treatment with vehicle (1% Teen 80, 5 mL/Kg;* p.o.*) or SLG (100, 200, or 400 mg/Kg;* p.o.*), according to Mothana et al. (2012) [22] with modifications. The time (in seconds) which elapsed until a typical distress reaction (jump out the surface or a hind paw flinch) was recorded as the latency of pain response. The cutoff time was set at 30 seconds to avoid tissue damage.

#### 2.6.4. Open Field Test

The spontaneous locomotor activity was evaluated in rats treated with SLG using the open field test, as previously described [23]. The open field consisted in a wooden box (40 × 50 × 60 cm), whose floor was divided into 9 equal squares. One hour after the treatment with vehicle (1% Teen 80, 5 mL/Kg;* p.o.*) or extract (100, 200, or 400 mg/Kg;* p.o.*), animals (*n* = 6–8 per group) were placed into the apparatus and the number of squares crossed with the four paws was counted for 5 minutes. Right after the open field, the same animals were also tested in the hot plate test as described previously (Section 2.6.3).

### 2.7. Analysis of the Possible Antinociceptive Mechanisms of Action of SLG

#### 2.7.1. Involvement of Opioid System: Pretreatment with Naloxone

To evaluate the possible participation of the opioid system in the antinociceptive activity of SLG, male Wistar rats were pretreated with naloxone (1 mg/Kg,* i.p.*) or saline (equivalent volume,* i.p.*). After 30 min, the experimental groups received the extract (at a dose of 200 mg/Kg* p.o.*) or vehicle (1% Teen 80, 5 mL/Kg). The nociceptive response (number of flinches) to the formalin injection was quantified during 1 h after the administration of SLG treatment, as described above.

#### 2.7.2. Involvement of Opioid System: Analysis of Intestinal Motility

To investigate whether the SLG, as morphine acting on *μ* opioid receptors, could induce a reduction of the oral-cecal transit, the intestinal motility assay model according to Stickney and Northup (1959) [24] was chosen. For this, animals were divided into five experimental groups (*n* = 6–8) and treated with vehicle (1% Teen 80, 5 mL/Kg); SLG (100, 200, or 400 mg/Kg,* p.o*.); or morphine (positive control; 5 mg/Kg,* s.c.*). After one hour of the treatments with extract or vehicle, or 30 min after morphine treatment, all groups received phenol red (1.5 mL, 0.05% in carboxymethylcellulose,* p.o*.). After 15 minutes of pigment administration, all animals were euthanized, the intestines were removed, and the displacement of the pigment was measured with a ruler. The percentage of intestinal motility inhibition was calculated according to the following formula: Intestinal motility inhibition (%) = (displacement of the pigment/total length of the small intestine) × 100.

#### 2.7.3. Involvement of TRPA1 Receptors

Since TRPA1 receptors have been related as the main site of formalin-induced nociceptive response [25], the possible role of TRPA1 receptors in the antinociceptive effect of SLG was evaluated in an independent experiment. For this, animals (*n* = 6–8 per group) were treated with SLG (200 mg/Kg,* p.o.*) or vehicle (1% Teen 80, 5 mL/Kg) and, one hour later, they received a subcutaneous injection of TRPA1 receptor agonist mustard oil (20 *μ*L, 0.5% diluted in corn oil) or an equivalent volume of corn oil into the dorsal right paw. Then, the number of flinches was counted cumulatively for 30 minutes, divided in 5-minute intervals.

### 2.8. Studies on Antioxidant Activity

#### 2.8.1. Scavenging Activity of 1,1-Diphenyl-2-picrylhydrazyl (DPPH) Radical

The radical scavenging activity of EtOH crude extract (SLG) and organic extracts on DPPH free radicals was measured using a qualitative and a quantitative method. The qualitative method described by Conforti et al. (2002) [26] is based on the thin layer chromatography (TLC) of the samples which is revealed with a DPPH solution. The samples which are positive controls (ascorbic acid and rutin) were diluted in methanol (3 mg/mL) and a TLC was performed. The revelation was performed with a 0.2% (w/v) DPPH MeOH solution. After 30 min, the samples which presented a yellow color were considered as a potential antioxidant and were submitted to a quantitative assay according to Ao et al. (2011) [27] with modifications. Reaction mixtures containing test samples diluted in various concentrations (2.5 mL) and a 300 *μ*M DPPH solution (1 mL) were left in tubes to react at room temperature for 30 min. The absorbance values were measured at 518 nm against the solvent without DPPH. The percentage of DPPH scavenging effect was determined and IC_50_ values denote the concentration of sample required to scavenge 50% of DPPH free radicals. Ascorbic acid and rutin were used as positive control.

#### 2.8.2. Total Phenolic Content

The total phenolic content of the SLG and organic extracts was determined through the Folin-Ciocalteu method [28] with modifications. Briefly, stock solutions of the extracts were prepared at 1 mg/mL concentration. Aliquots from the stock solutions (320 *μ*L of SLG, CHCl_3_, and EtOH-H_2_O extracts, 640 *μ*L of* n*-hexane, and 160 *μ*L of EtOAc extracts) were added to 200 *μ*L of Folin-Ciocalteu reagent and distilled water to 4.0 mL. The mixture was shaken and then 400 *μ*L of 10% Na_2_CO_3_ was added and shaken again. After 30 min, the absorbance was measured at 760 nm. The obtained data were confronted to a gallic acid calibration curve.

#### 2.8.3. Thiobarbituric Reactive Substances (TBARS)

This method was performed in order to evaluate the influence of SLG and organic extracts on the lipid peroxidation inhibition according to Cox et al. (2005) [29]. A 5% (w/v) egg yolk solution in sodium dodecyl sulfate (0.55% in distilled water) was used as a source of lipids. The lipid solution (0.5 mL) was added to the extracts (0.1 mL, 3 mg/mL), 2,2′-azo-bis-2-amidinopropanochloride (50 *μ*L, 0.035 M), acetic acid (20%, 1.5 mL), distilled water (0.4 mL), and thiobarbituric acid (0.4%, 1.5 mL). After 60 minutes at 95°C the organic phase was extracted with ButOH (1.5 mL) and the absorbance was measured at 532 nm. The antioxidant activity was expressed as percent of reduction of peroxidation (RP%) calculated using the following formula: (peroxidation indicator value without antioxidant) − (peroxidation indicator with antioxidant)/(peroxidation indicator value without antioxidant) × 100. A control containing no antioxidant was 0% and BHT was used as standard.

### 2.9. Statistical Analyses

All results were presented as mean ± SEM. The statistical significance of differences between groups was performed by one-way ANOVA, followed by Newman-Keuls test for behavioral tests and by Tukey test for DPPH and TBARS assays. *p* < 0.05 was considered as indicative of significance.

## 3. Results

### 3.1. Phytochemical Investigation

**Figure 1:**
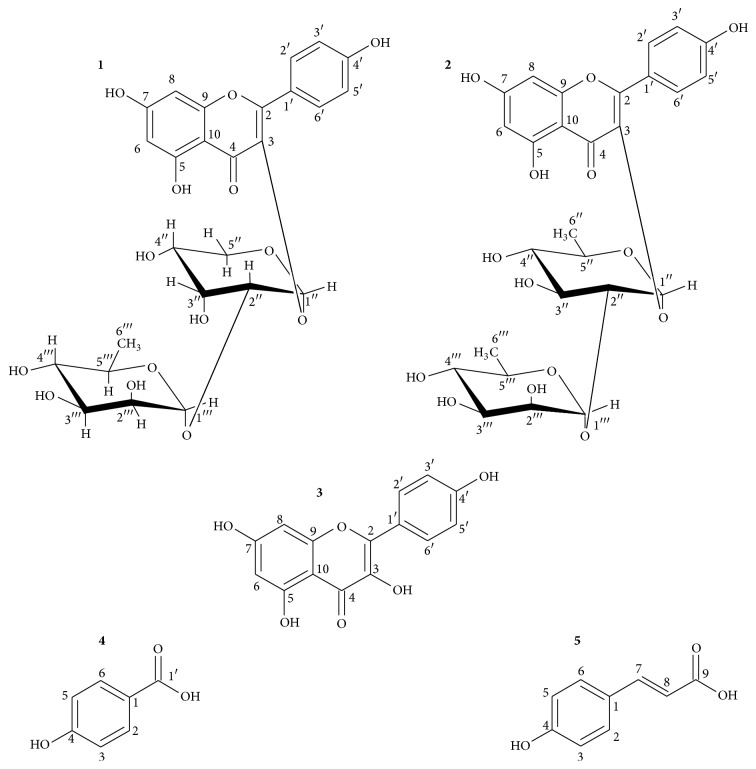
Compounds identified in* Smilax larvata*.

The phytochemical screening of extracts of* S. larvata* indicated the presence of flavonoids, coumarins, steroids, triterpenes, alkaloids, tannins, and amino groups. Therefore, SLG was submitted to several chromatographic purification processes [see Figure S11 in Supplementary Material available online at http://dx.doi.org/10.1155/2016/9894610], leading to the identification of two glycosylated flavonoids (**1** and** 2**, Figure 1) as well as an aglycone flavonoid (**3**) and two* p*-phenolics (**4** and** 5**). The structures of compounds were established on the basis of extensive analysis of ^1^H, ^13^C, HMBC, and HSQC NMR experiments as well as comparing with literature data [Figures S1–S10 in supplementary data]. In this way, compounds** 1** to** 5** were identified as drabanemoroside [30], kaempferol 3-*O*-*α*-L-rhamnopyranosyl(1→2)-*α*-L-rhamnopyranoside [31], kaempferol [32],* p*-hydroxybenzoic acid [33], and* p*-coumaric acid [34], respectively.

### 3.2. Pharmacological Studies

#### 3.2.1. Effect of SLG on Carrageenan-Induced Paw Edema in Rats

**Figure 2:**
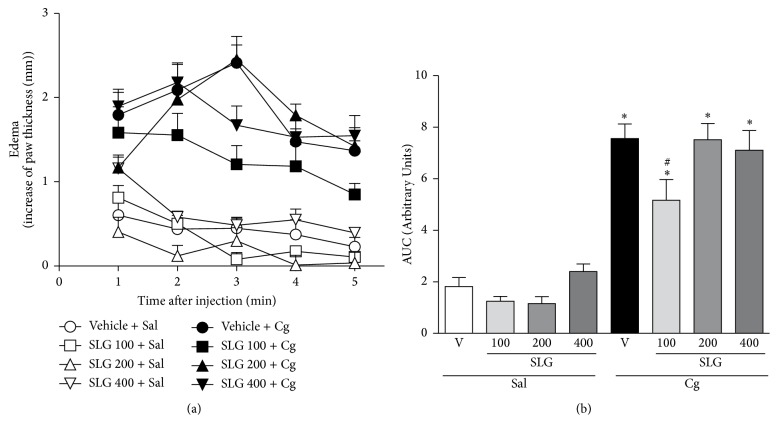
Effect of SLG treatment on carrageenan- (Cg-) induced edema in rats. (a) Data are expressed as the mean ± SEM of the increasing in the paw thickness (mm), calculated by subtracting the paw thickness measured at 1, 2, 3, 4, and 5 h after Cg or saline (Sal) injection to the initial paw thickness (basal). (b) Total edema during the 5 h was calculated as area under the time course curves (AUC). ^*∗*^ *p* < 0.05 when compared to saline + vehicle- (V-) treated group; ^#^ *p* < 0.05 when compared to vehicle + Cg-treated group.

As shown in Figures 2(a) and 2(b), in the vehicle-treated group, the maximum paw edema formation was observed 2 to 3 h after Cg injection (Figure 2(a)). Pretreatment with SLG only at a dose of 100 mg/Kg significantly attenuated the paw edema formation by 32% when compared to vehicle plus Cg group (Figure 2(b)).

#### 3.2.2. Effect of SLG on Formalin-Induced Nociception in Rats

**Figure 3:**
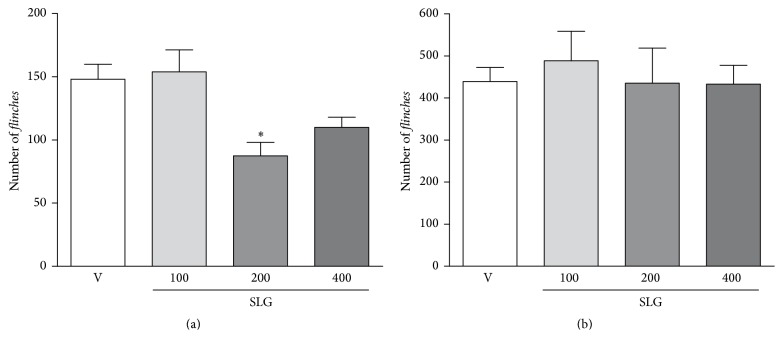
Effect of SLG treatment on formalin-induced paw flinches in rats. Data are expressed as the mean ± SEM of the number of paw flinches during the first (a) or the second phase (b) of the formalin test in rats treated with vehicle (V) or SLG. ^*∗*^ *p* < 0.05 when compared to vehicle (V).

When compared to vehicle-treated group (Figures 3(a) and 3(b)), the animals treated with SLG (only at a dose of 200 mg/Kg) exhibited a significant decrease in the number of formalin-induced flinches by 41% during the first phase of the test (Figure 3(a)). However, the treatment with SLG (at doses of 100, 200, or 400 mg/Kg) was not able to induce a significant effect over the formalin-induced flinches during the second phase of the test (*p* > 0.05; Figure 3(b)).

#### 3.2.3. Effect of SLG on Acetic Acid-Induced Writhing in Mice

**Figure 4:**
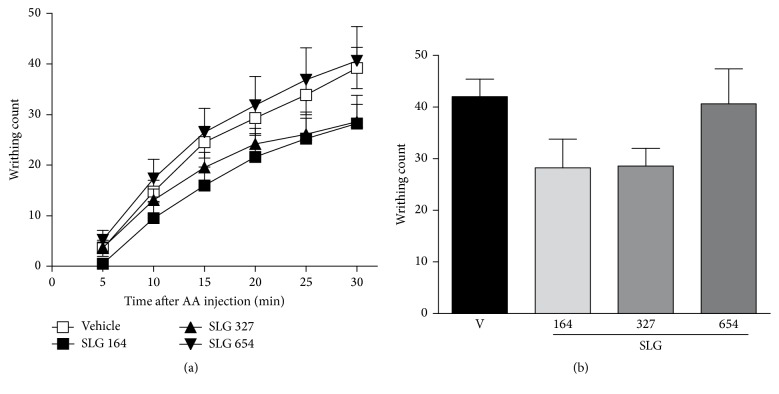
Effect of SLG treatment on writhings induced by acetic acid (AA) in mice. (a) Number of writhings induced by acetic acid during 30 min, counted cumulatively every 5 minutes. (b) Total writhing response during the 30 min. Data are expressed as the mean ± SEM of 10–15 mice/experimental group.

As observed in Figures 4(a) and 4(b), the treatment with SLG (at doses of 164, 327, or 654 mg/Kg) did not present a statistically significant antinociceptive effect in acetic acid-induced writhing in mice (*p* > 0.05).

#### 3.2.4. Effect of SLG on Hot Plate and Open Field Tests

**Figure 5:**
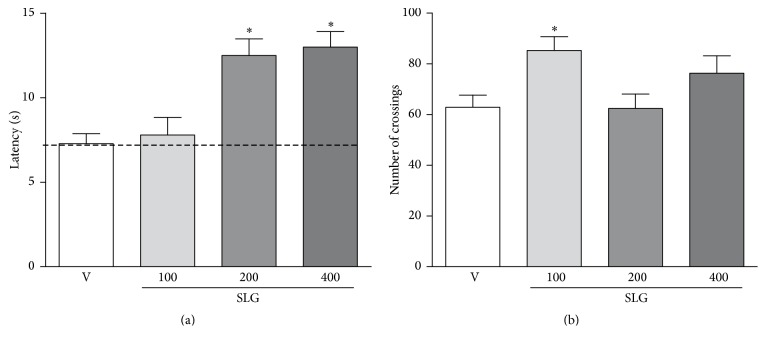
Effect of SLG treatment on hot plate latency (a) and in number of crossings during the open field test (b) in rats. Data are expressed as the mean ± SEM. ^*∗*^ *p* < 0.05 when compared to vehicle- (V-) treated group.

As demonstrated in Figure 5(a) the treatment with SLG (at doses of 200 or 400 mg/Kg, but not at a dose of 100 mg/Kg) significantly increased the hot plate reaction time by 72% and 78%, respectively, when compared to vehicle-treated rats. In the open field test, the treatment with SLG (at doses of 200 or 400 mg/Kg) did not significantly affect the number of crossings in the open field when compared to vehicle-treated group (Figure 5(b)). However, the SLG treatment at a dose of 100 mg/Kg significantly increased the number of crossings in the open field by 35%.

#### 3.2.5. Study of Possible Antinociceptive Mechanism of SLG: Involvement of Opioid System

**Figure 6:**
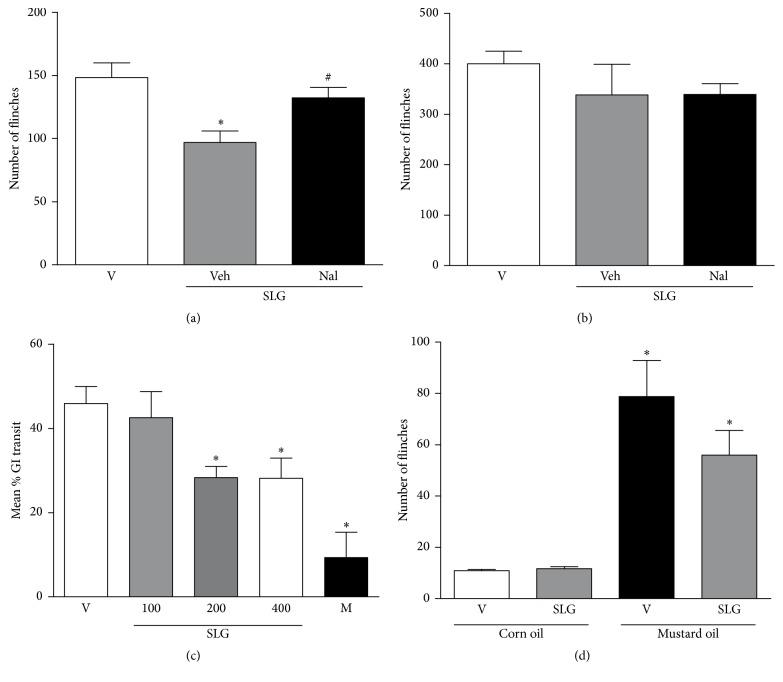
Antinociceptive mechanism of SLG. (a) and (b) represent the mean ± SEM of the number of paw flinches during the first (a) or the second phase (b) of the formalin test in rats pretreated with saline (Veh) or naloxone (Nal) plus SLG. ^#^ *p* < 0.05 when compared to vehicle (V) + SLG 200-treated group. ^*∗*^ *p* < 0.05 when compared to vehicle- (V-) treated group. (c) represents the effect of SLG treatment on intestinal motility in rats. Data are expressed as the mean ± SEM of percentage of intestinal motility inhibition in animals treated with vehicle (V), SLG, or morphine (M). ^*∗*^ *p* < 0.05 when compared to vehicle- (V-) treated group. (d) Effect of SLG treatment (200 mg/Kg) on mustard oil-induced paw flinches in rats. Data are expressed as the mean ± SEM of the number of paw flinches observed during 30 min after mustard or corn oil treatment. ^*∗*^ *p* < 0.05 when compared to vehicle (V) + corn oil-treated group.

As shown in Figure 6(a), the pretreatment with a nonselective opioid receptor antagonist naloxone completely reversed the antinociceptive effect of SLG (at a dose of 200 mg/Kg) during the first phase of the formalin test. However, as observed previously (Figure 3(b)) the treatment with SLG (at same dose) did not significantly alter the number of formalin-induced flinches during the second phase of the test (Figure 6(b)). In the same way, the pretreatment with naloxone did not induce a significant effect in the number of flinches during the second phase of the test when compared to vehicle-treated group (Figure 6(b)).

In the intestinal motility assay, as demonstrated in Figure 6(c), while the treatment with morphine suppressed the gastrointestinal transit by 80% when compared to vehicle-treated group, the treatment with SLG (at doses of 200 or 400 mg/Kg, but not with 100 mg/Kg) significantly reduced the intestinal motility by 45% and 46%, respectively.

#### 3.2.6. Study of Possible Antinociceptive Mechanism of SLG: Involvement of TRPA1 Receptors

As expected, the intraplantar mustard oil injection induced a marked increase of nociceptive responses (evaluated as a number of paw flinches) when compared to corn oil-treated group (Figure 6(d)). The treatment with SLG (200 mg/Kg) did not significantly affect the mustard oil-induced nociceptive responses when compared to vehicle-treated group (*p* > 0.05).

#### 3.2.7. Antioxidant Activity Investigation

In the DPPH tests, it was revealed that the EtOAc extract has a moderate antioxidant activity with IC_50_ 58.17 *μ*g/mL. On the other hand, the positive controls vitamin C and rutin showed IC_50_ of 4.3 and 5.8 *μ*g/mL, respectively. The EtOAc extract showed the highest phenol content (375 mg GAE/g of dry extract) as compared to CHCl_3_ (271 mg GAE/g of dry extract) and EtOH-H_2_O extracts (139 mg GAE/g of dry extract).* S. larvata* extracts were able to inhibit lipid peroxidation, the CHCl_3_ being the most effective extract, followed by SLG and* n*-hexane extracts, which reduced the percentage of lipid peroxidation by 78%, 59%, and 42%, respectively. Interestingly, these values are very similar to those obtained using the positive control (BHT = 44%, Figure S12 in supplementary information).

## 4. Discussion

The main finding of this study is that the treatment with a crude ethanolic extract of* S. larvata* exerted a significant antinociceptive effect, which seems to involve the opioid system activation. This result is supported by both pharmacological data and phytochemical detection of alkaloids.

The qualitative phytochemical screening results are in agreement with previous works on* Smilax* species [2]. After chromatographic purification processes we identified three flavonoids, (**1**) drabanemoroside, (**2**) kaempferol 3-*O*-*α*-L-rhamnopyranosyl(1→2)-*α*-L-rhamnopyranoside, and (**3**) kaempferol, and two phenolic compounds, (**4**)* p*-hydroxybenzoic acid and (**5**)* p-*coumaric acid, in EtOAc [30–34]. The kaempferol (**3**) is widely distributed on vegetable kingdom, including the genus* Smilax* [35]. Studies reported that kaempferol exhibited an antioxidant activity, acting through multiple mechanisms of action, including scavenging of superoxide anions, hydroxyl and peroxynitrite radicals, leading to a significant prevention of lipid peroxidation. This property could be related to its anti-inflammatory activity, beyond its capability of cyclooxygenase 2 inhibition [36]. The phenylpropanoid* p*-coumaric acid (**5**) is a precursor in the biosynthesis of a wide range of natural products. Previous studies indicated that* p*-coumaric acid presented an antioxidant activity in vitro, and when added to rats diet, it exerted an antioxidant effect in colonic mucosa [37]. Regarding the* p*-hydroxybenzoic acid (**4**), the biological activities reported include antifungal, antimutagenic, and estrogenic activities [38]. Finally, for the first time in the literature, our study identified the glucoside flavonoids drabanemoroside (**1**) and kaempferol 3-*O*-*α*-L-rhamnopyranosyl(1→2)-*α*-L-rhamnopyranoside in the genus* Smilax*. There are few studies on drabanemoroside biological activities, and some reports about its antitumoral potential are contradictories [30, 39]. Regarding kaempferol 3-*O*-*α*-L-rhamnopyranosyl(1→2)-*α*-L-rhamnopyranoside, no studies about its biological activities have been described yet.

Since the tea of aerial parts of* Smilax larvata* Griseb. has been used as anti-inflammatory, the first in vivo study aimed to validate this use testing the effect of SLG in the carrageenan-induced paw edema model. As observed in Figure 2, the treatment with SLG (only at a dose of 100 mg/Kg) induced a moderate anti-inflammatory activity in this test. Carrageenan, a usual phlogistic agent, has been extensively used as an animal model for detection of the anti-inflammatory effects of drugs and plants [28, 40] since it causes noteworthy inflammatory responses, including paw edema. In the present study, even this modest anti-inflammatory activity of SLG may be explained by the presence of polyphenols, flavonoids as kaempferol, and coumarins, which have been reported as anti-inflammatory agents [41].

At the next set of in vivo experiments, we aimed to evaluate the possible antinociceptive effect of SLG since pain is a frequent symptom of the inflammatory process and drugs with anti-inflammatory effect very often present also analgesic properties [42]. Firstly, we tested the effect of SLG in the formalin test, since this test has been described as a useful tool for initial screening of drugs with both anti-inflammatory and antinociceptive properties [26]. This method is characterized by a biphasic pattern of nociceptive behaviors, the first phase being described as a direct consequence of a chemical stimulation of nociceptors by formalin, that is, the neurogenic phase which lasts five minutes after the formalin injection. The second phase is attributable to a local inflammatory reaction responsible for sensitization of primary and spinal sensory neurons with a subsequent activation of the nociceptors, starting 15 minutes after formalin and lasting for the following 45 minutes [43]. The significant antinociceptive effect of SLG treatment during the first phase of formalin test (Figure 3(a)) and the absence of effect of SLG during the second phase of formalin test (Figure 3(b)) diverge from the ethnopharmacological use of* S. larvata* as anti-inflammatory. However, these data suggest a potential analgesic effect of SLG, which was further investigated in the next set of experiments using the acetic acid-induced writhing test. As shown in Figure 4, the treatment with SLG did not induce a significant reduction of the acetic acid-induced writhings. Our hypothesis to explain this absence of SLG effect is due to the fact that the abdominal constriction induced by acetic acid is related to an increase of synthesis/release of multiple inflammatory mediators, such as prostaglandins, leukotrienes, 5-HT, histamine, and kinins [44] which, in turn, chemically sensitize the nociceptors [45]. Then, our theory is that the SLG effect was not able to counteract the nociceptive effect of acetic acid since it does not interfere with the release and/or with the action of these mediators. Taken together, the absence of SLG effect in carrageenan-induced paw edema and in acetic acid-induced writhings suggests that the effect of SLG may be associated with a more analgesic than anti-inflammatory effect. These apparently contrasting data were further explored using a hot plate test, a model that discriminates pain in the central and peripheral components. In this test, treatment with SLG at the doses of 200 and 400 mg/Kg significantly raised the thermal latency of pain responses. Since it has been observed that the pain response to a thermal stimulus in hot plate is selective for centrally acting analgesics, while the peripherally acting agents are inactive [46], these results indicate a possible central antinociceptive effect of SLG.

To exclude possible muscle relaxant or sedative effects, which could alter motor performance of the animals and result in false antinociceptive results, animals were treated with SLG and submitted to the open field test. It was observed that only the treatment with SLG at a dose of 100 mg/Kg significantly increases the number of crossings in the open field, indicating an increase of locomotor activity and exploration when compared to vehicle-treated group [43]. The stimulant effect in the open field led us to disregard this dose as an effective dose in the studies about the possible antinociceptive mechanisms of action of SLG.

Regarding the possible mechanisms of action of SLG, we firstly have tested the opioid system since our results obtained in the hot plate test (Figure 5) and the presence of alkaloids detected on phytochemical screening support this hypothesis. To test it, an independent group of rats was pretreated with naloxone, a nonselective antagonist of opioid receptors, before the SLG treatment, and then the formalin test was performed. It was observed that the pretreatment with naloxone completely prevented the antinociceptive effect of SLG (200 mg/Kg) during the first phase (neurogenic) of the formalin test, indicating the opioid system involvement. In order to confirm the opioid-like action, the effect of SLG over the gastrointestinal motility was also tested, since the opioids capacity of inhibiting the intestinal motility has been well established, mainly due to binding to *μ* opioid receptors [47]. Interestingly, the same doses of SLG that induced an evident increase of reaction time in the hot plate test (200 or 400 mg/Kg) induced a clear reduction of intestinal motility (Figure 6(b)), corroborating to the hypothesis that the antinociceptive effect of the extract may be related to the opioid system activation.

Finally, it was verified whether the antinociceptive effect of SLG observed in the formalin test could be related to its effect over TRPA1 receptors. This suggestion was tested given that TRPA1 receptors have been related as the main site of formalin-induced nociceptive response [25]. We observed that the direct nociceptive responses induced by mustard oil, a selective TRPA1 agonist, were not significantly affected by the SLG treatment (Figure 6(d)), suggesting that the antinociceptive effect of SLG does not seem to be related to TRPA1 antagonism.

This significant antinociceptive effect of SLG may be related to the presence of the compounds phytochemically characterized in this study. In this way, it is important to highlight that the drabanemoroside and the kaempferol 3-*O*-*α*-L-rhamnopyranosyl(1→2)-*α*-L-rhamnopyranoside present the flavonol kaempferol as the aglycone. Previous studies have already observed an antinociceptive activity of other glycosides of this flavonol, mainly over the inflammatory pain [48, 49]. The antioxidant and anti-inflammatory activities of the* p*-hydroxybenzoic acid have already been described previously [50, 51]. In relation to its potential antinociceptive effect, it is interesting to note that the* p*-hydroxybenzoic acid is an isomer of the salicylic acid, a molecule widely used for pain treatment [52]. Furthermore, kaempferol treatment has been associated with an increase of the latency time in the hot plate test and also an increase of the mechanical threshold in diabetic neuropathic pain in mice. This antinociceptive effect was attributed by the authors to its antioxidant activity [48]. Finally, it is noteworthy to mention that the presence of alkaloids (detected in the qualitative chemical screening) may be related to the opioid effects observed in the current study, that is, the antinociceptive effect over formalin-induced flinches reverted by naloxone (Figure 6(a)); the increase in thermal latency time in the hot plate (Figure 5(a)); and the increase in gastrointestinal motility (Figure 6(c)). Thus, our hypothesis is that these and other compounds may be acting synergistically for the biological activities of SLG, including its antioxidant activity.

Since the oxidative/nitrosative stress has been extensively associated with inflammation and different types of pain [22], substances with antioxidant properties have been proposed as adjuvant drugs to treat inflammation and pain. For this reason, in the next set of experiments, the possible antioxidant effect of SLG was also investigated. Firstly, the DPPH assay, one of the most common methods to investigate the ability of compounds to act as free radical scavengers [29], was used combined with thin layer chromatography (TLC) analyses. The results of TLC and quantitative DPPH assays indicate that* S. larvata* presents, in general, low amounts of compounds with property of free radical scavenging. The antioxidant activity of the EtOAc extract can be due to the presence of phenolic compounds, which presents antioxidant activity by donating hydrogen atoms or electrons and thereby scavenges free radicals [22]. These findings are in accordance with the total phenolic content, which showed a low amount of these compounds in* S. larvata* extracts, comparing with other* Smilax* species [2]. However, CHCl_3_, SLG, and* n*-hexane extracts significantly reduced the lipid peroxidation. The phytochemical screening revealed the presence of low polarity compounds, such as coumarins and alkaloids, both inhibitors of lipid peroxidation [53, 54]. These results are in accordance with previous studies on antioxidant activity of* Smilax* species extracts, which presented diverse mechanisms of action, such as quenching of superoxide anion, lipid peroxidation inhibition, deoxyribose degradation inhibition, and reducing power [29, 55]. Beyond the alkaloids, the flavonoids present in SLG may be contributing in a synergic way to the antinociceptive activity observed [56].

## 5. Conclusion

The phytochemical investigation of* Smilax larvata* resulted in kaempferol,* p*-hydroxybenzoic acid,* p*-coumaric acid, and two glucoside flavonoids described for the first time in this genus, drabanemoroside and kaempferol 3-*O*-*α*-L-rhamnopyranosyl(1→2)-*α*-L-rhamnopyranoside. Moreover, we observed that the* S. larvata* crude ethanolic extract has a significant antinociceptive action, which seems to be associated with opioid system activation without TRPA1 receptor involvement. Consistent with the phytochemical study, these findings suggest that the alkaloids detected in the ethanolic extract appear to be responsible for the antinociceptive activity, and the phenolic compounds isolated may be acting synergistically. The antioxidant investigation corroborates these results, since lipid peroxidation inhibition is an antioxidant mechanism related to a low polarity compound action, such as alkaloids.

## Supplementary Material

Figure S1 – Full ^1^H NMR spectrum of compounds 3, 4 and 5 (MeOD/TMS, 600.13 MHz, 30°C).
Figure S2 – Expansion of ^1^H NMR spectrum of compounds 3, 4 and 5, showing the aromatic signals and its integrals (MeOD/TMS, 600.13 MHz, 30°C).
Figure S3 – Expansion of one-bond ^1^H-^13^C correlation map from HSQC NMR experiment at 600.13 and 150.9 MHz, highlighting correlations for aromatic hydrogens of compounds 3, 4 and 5 (MeOD/TMS, 30°C).
Figure S4 – Expansion of long range ^1^H-^13^C correlation map from HMBC NMR experiment at 600.13 and 150.9 MHz, highlighting correlations for aromatic hydrogens of compounds 3, 4 and 5 (MeOD/TMS, 30 °C).
Figure S5 – Full ^1^H NMR spectrum of compound 1 (MeOD/TMS, 200.13 MHz, 30 °C).
Figure S6 – Full ^13^C NMR spectrum of compound 1 (MeOD/TMS, 200.13 MHz, 30 °C).
Figure S7 – Expansion of one-bond ^1^H-^13^C correlation map from HSQC NMR experiment at 600.13 and 150.9 MHz, highlighting correlations for hydrogens of compound 1 (MeOD/TMS, 30 °C).
Figure S8 – Expansion of one-bond ^1^H-^13^C correlation map from HMBC NMR experiment at 600.13 and 150.9 MHz, highlighting correlations for hydrogens of compound 1 (MeOD/TMS, 30 °C).
Figure S9 – Full ^1^H NMR spectrum of compound 2 (MeOD/TMS, 200.13 MHz, 30 °C).
Figure S10– Full ^13^C NMR spectrum of compound 2 (MeOD/TMS, 200.13 MHz, 30 °C).
Figure S11 – Chromatogram of preparative high performance liquid chromatography separation of compounds 1 and 2, witch correspond to the signals at 31.5 and 37.5 minutes respectively.
Figure S12 – Percent of reduction of peroxidation (RP%). Crude ethanolic extract of *Smilax larvata* (SLG), *n*-hexane extract (n-hex), chloroform extract (CHCl_3_), ethyl acetate extract (EtOAc), hydroalcoholic extract (EtOH-H_2_O). Data are expressed as the mean ± S.E.M. ∗∗∗*p*<0.001 when compared to control without antioxidant, #*p*<0.1 when compared to BHT.
