# Supplementary material for: Phytochemical and Antinociceptive, Anti-Inflammatory, and Antioxidant Studies of Smilax larvata (Smilacaceae)
Source: Evid Based Complement Alternat Med. 2016 Dec 22;2016:9894610. doi: 10.1155/2016/9894610 (PMC5214527; doi:10.1155/2016/9894610)
Supplement: Supplementary file 1 — Figure S1 – Full 1H NMR spectrum of compounds 3, 4 and 5 (MeOD/TMS, 600.13 MHz, 30°C). Figure S2 – Expansion of 1H NMR spectrum of compounds 3, 4 and 5, showing the aromatic signals and its integrals (MeOD/TMS, 600.13 MHz, 30°C). Figure S3 – Expansion of one-bond 1H-13C correlation map from HSQC NMR experiment at 600.13 and 150.9 MHz, highlighting correlations for aromatic hydrogens of compounds 3, 4 and 5 (MeOD/TMS, 30°C). Figure S4 – Expansion of long range 1H-13C correlation map from HMBC NMR experiment at 600.13 and 150.9 MHz, highlighting correlations for aromatic hydrogens of compounds 3, 4 and 5 (MeOD/TMS, 30 °C). Figure S5 – Full 1H NMR spectrum of compound 1 (MeOD/TMS, 200.13 MHz, 30 °C). Figure S6 – Full 13C NMR spectrum of compound 1 (MeOD/TMS, 200.13 MHz, 30 °C). Figure S7 – Expansion of one-bond 1H-13C correlation map from HSQC NMR experiment at 600.13 and 150.9 MHz, highlighting correlations for hydrogens of compound 1 (MeOD/TMS, 30 °C). Figure S8 – Expansion of one-bond 1H-13C correlation map from HMBC NMR experiment at 600.13 and 150.9 MHz, highlighting correlations for hydrogens of compound 1 (MeOD/TMS, 30 °C). Figure S9 – Full 1H NMR spectrum of compound 2 (MeOD/TMS, 200.13 MHz, 30 °C). Figure S10– Full 13C NMR spectrum of compound 2 (MeOD/TMS, 200.13 MHz, 30 °C). Figure S11 – Chromatogram of preparative high performance liquid chromatography separation of compounds 1 and 2, witch correspond to the signals at 31.5 and 37.5 minutes respectively. Figure S12 – Percent of reduction of peroxidation (RP%). Crude ethanolic extract of Smilax larvata (SLG), n-hexane extract (n-hex), chloroform extract (CHCl3), ethyl acetate extract (EtOAc), hydroalcoholic extract (EtOH-H2O). Data are expressed as the mean ± S.E.M. ∗∗∗p<0.001 when compared to control without antioxidant, #p<0.1 when compared to BHT. [file 9894610.f1.doc]

**Supporting information**

**1. NMR data**


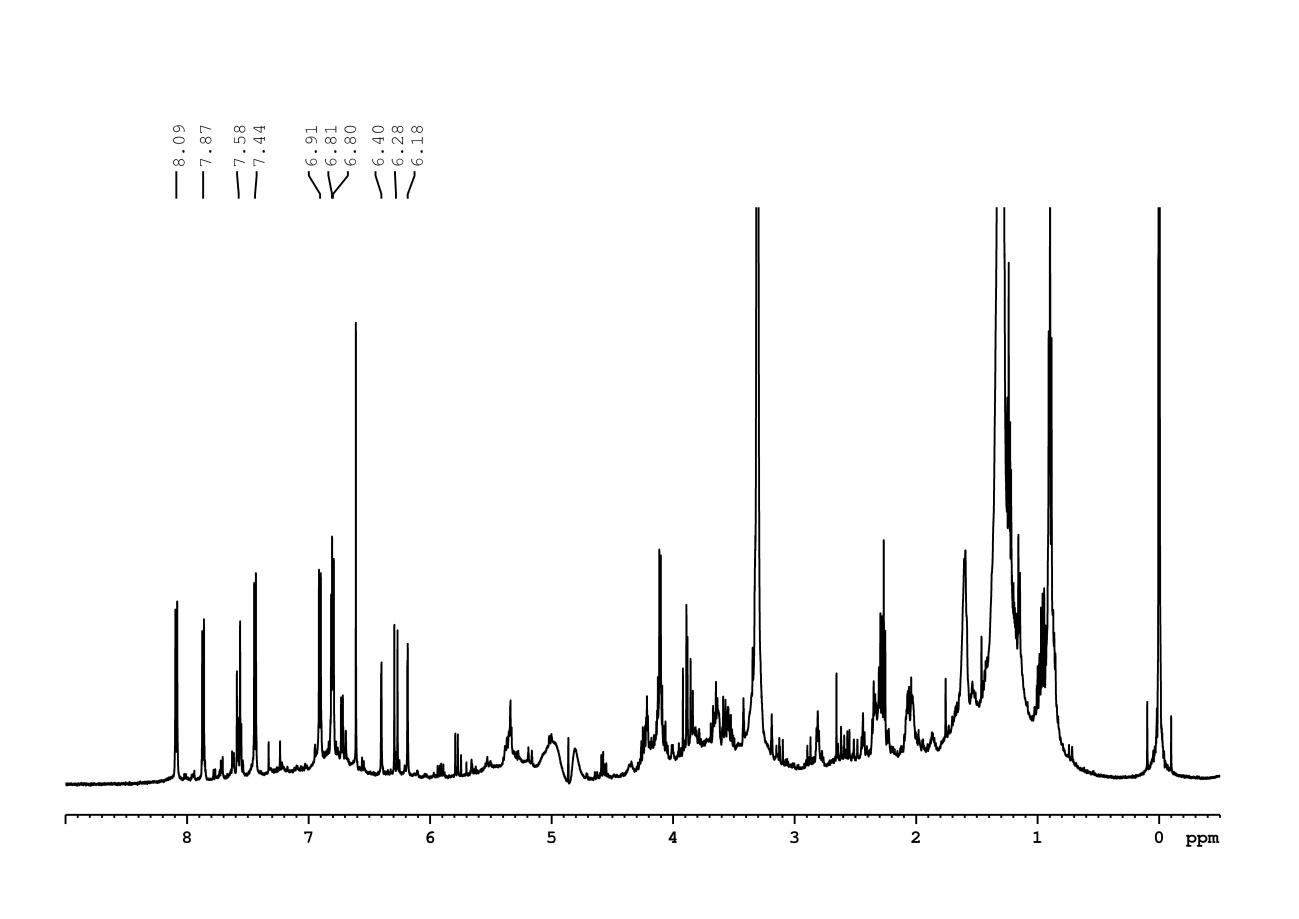


**Figure S1**– Full ^1^H NMR spectrum of compounds 3, 4 and 5 (MeOD/TMS, 600.13 MHz, 30°C).


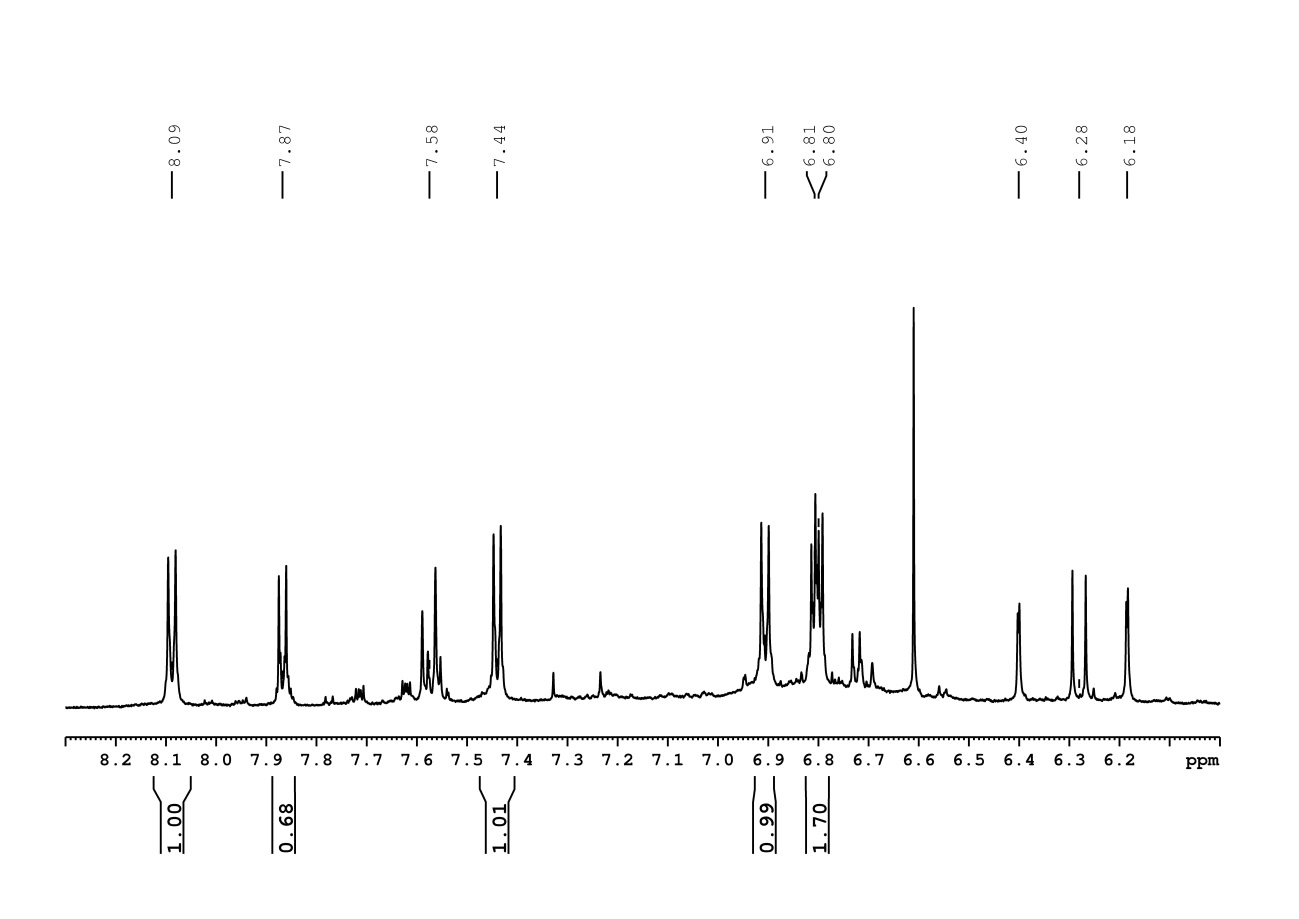


**Figure S2** – Expansion of ^1^H NMR spectrum of compounds 3, 4 and 5, showing the aromatic signals and its integrals (MeOD/TMS, 600.13 MHz, 30°C).


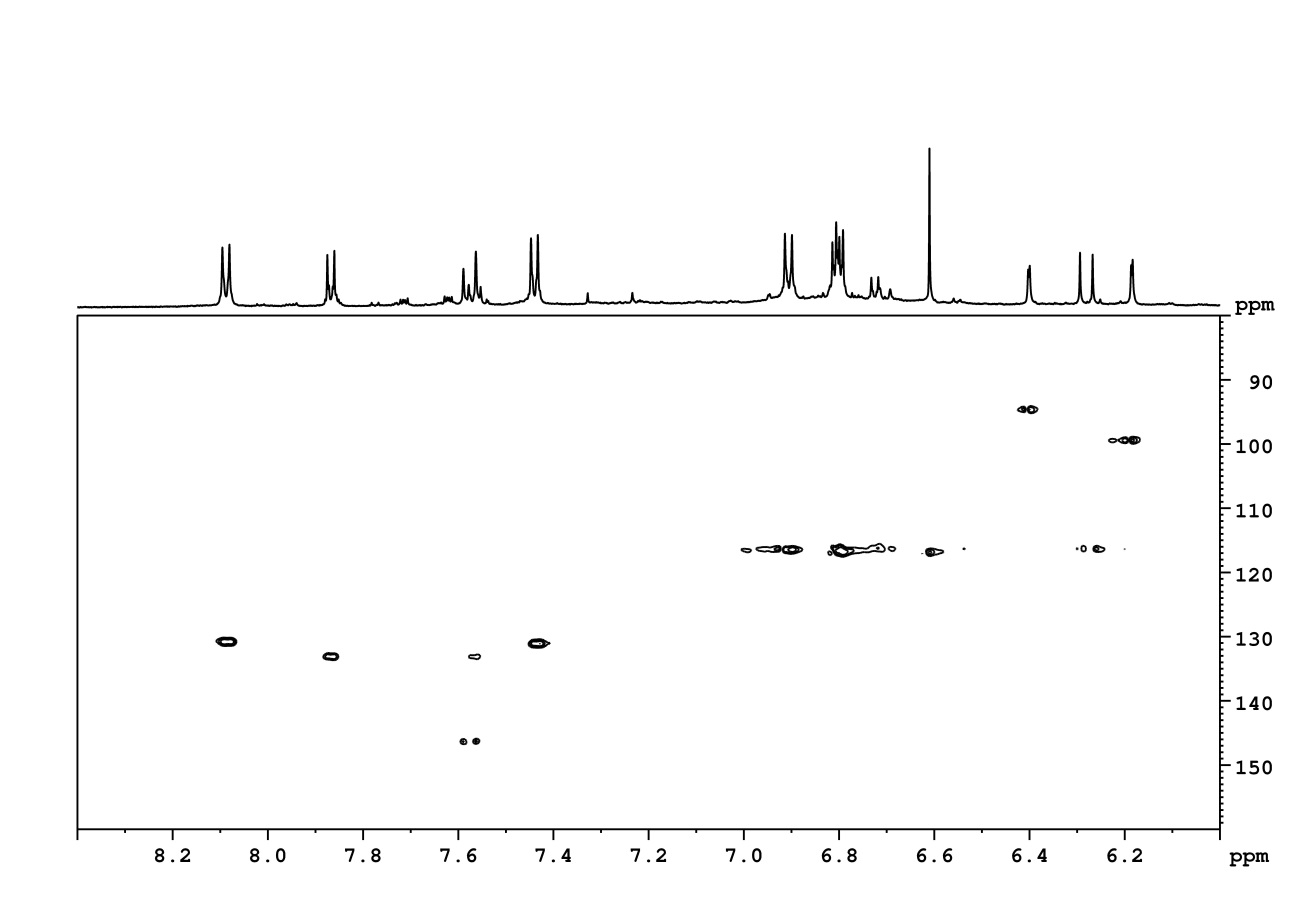


**Figure S3** – Expansion of one-bond ^1^H-^13^C correlation map from HSQC NMR experiment at 600.13 and 150.9 MHz, highlighting correlations for aromatic hydrogens of compounds 3, 4 and 5 (MeOD/TMS, 30°C).


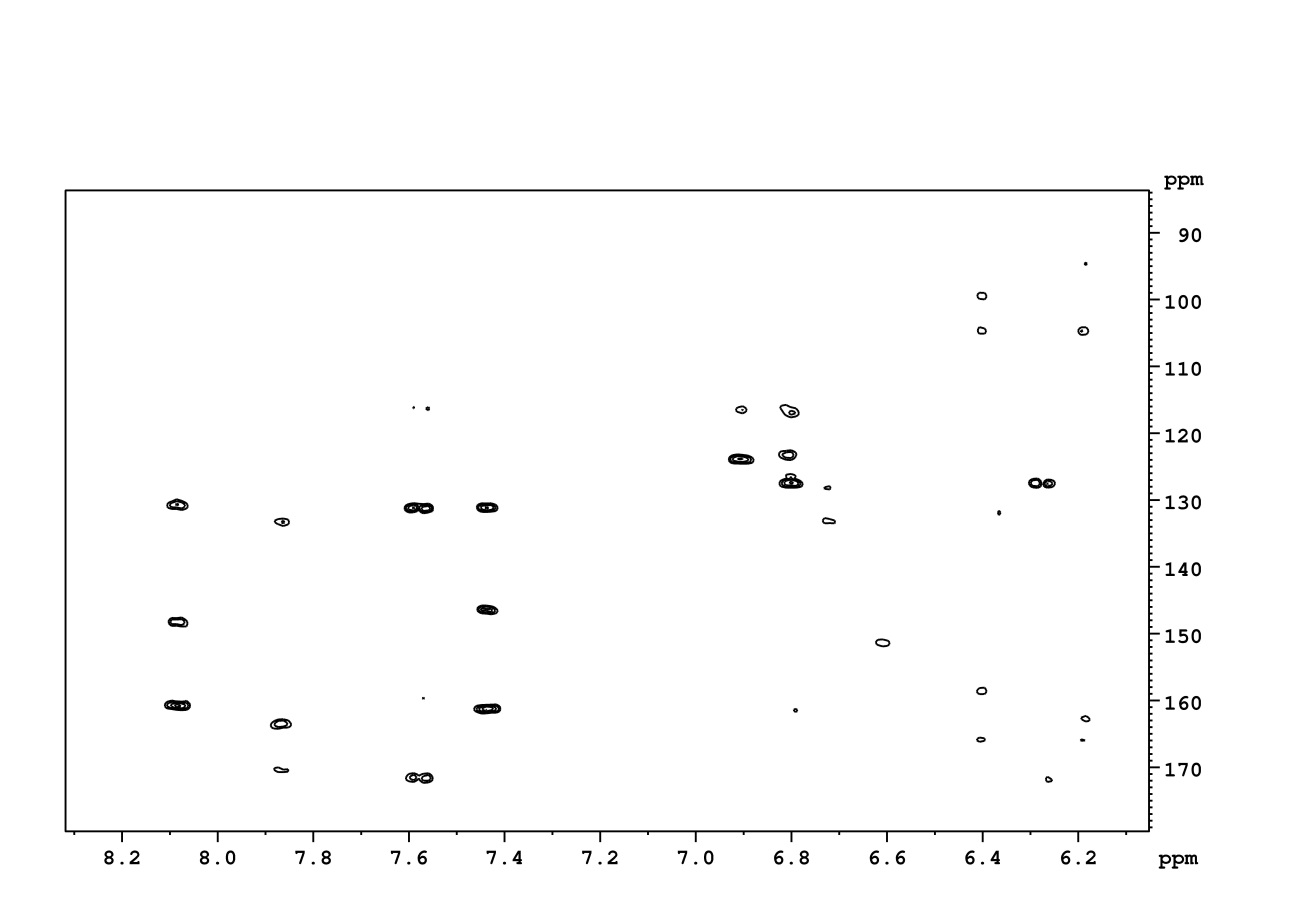


**Figure S4** – Expansion of long range ^1^H-^13^C correlation map from HMBC NMR experiment at 600.13 and 150.9 MHz, highlighting correlations for aromatic hydrogens of compounds 3, 4 and 5 (MeOD/TMS, 30 °C).


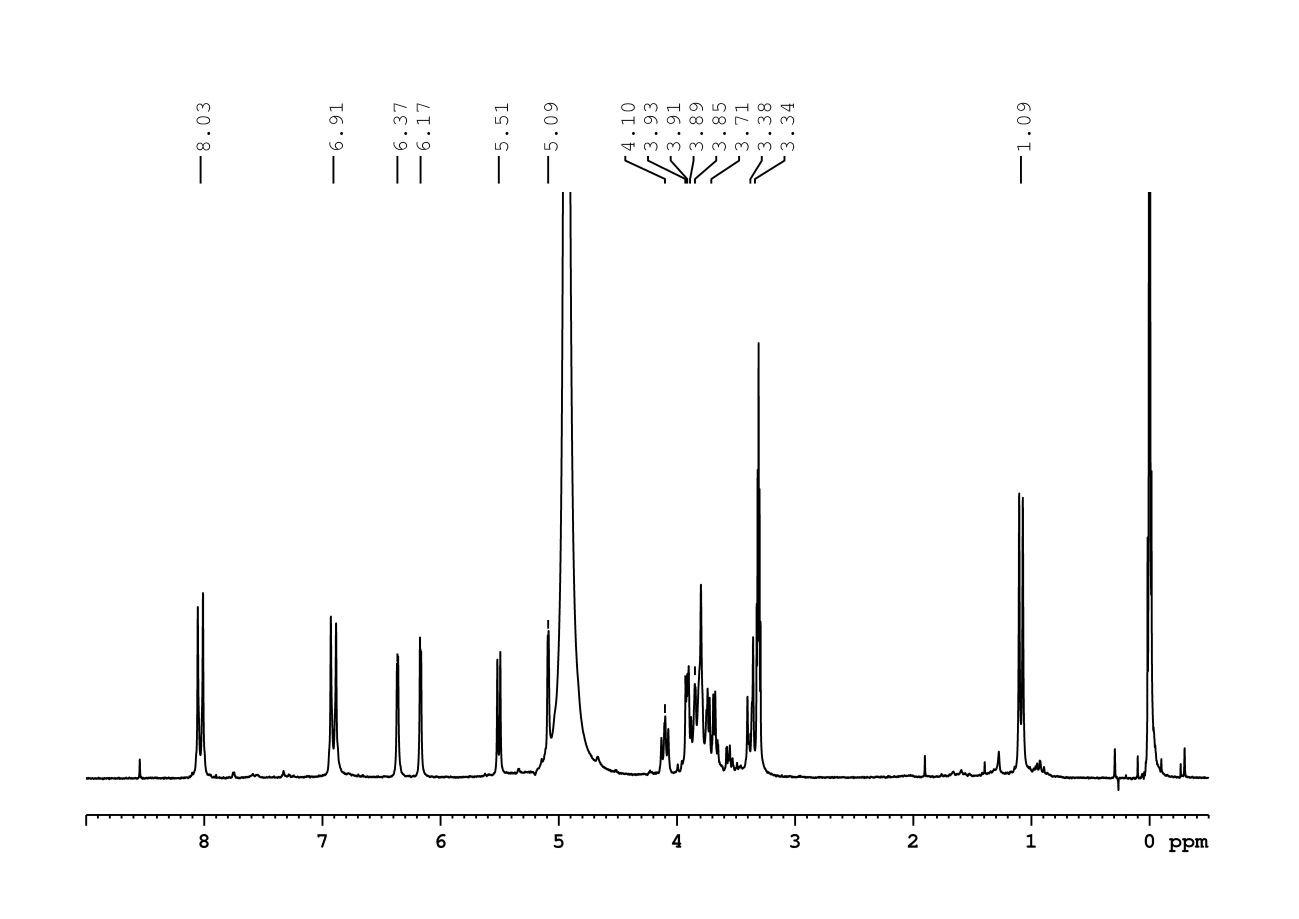


**Figure S5** – Full ^1^H NMR spectrum of compound 1 (MeOD/TMS, 200.13 MHz, 30 °C).


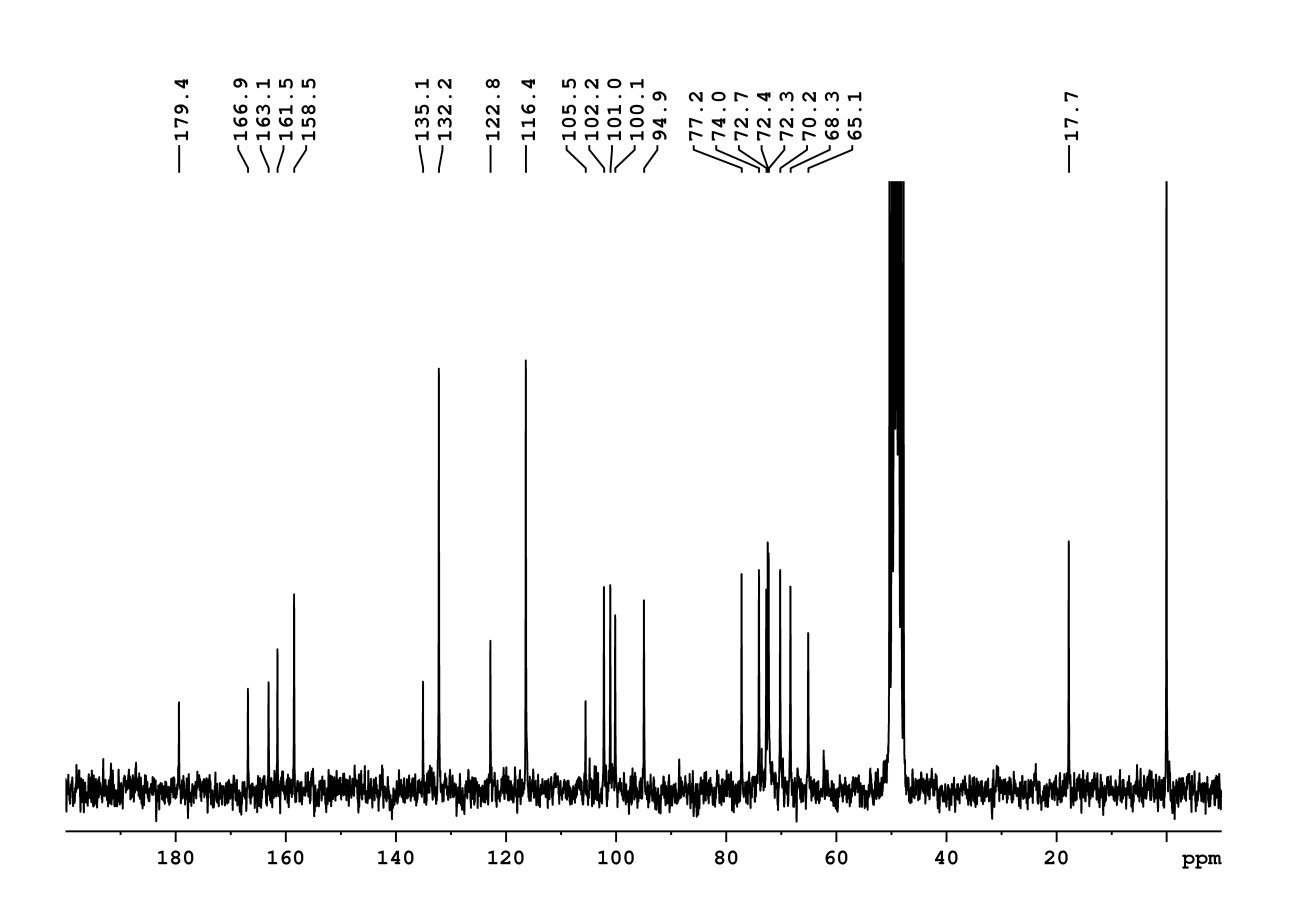


**Figure S6** – Full ^13^C NMR spectrum of compound 1 (MeOD/TMS, 200.13 MHz, 30 °C).


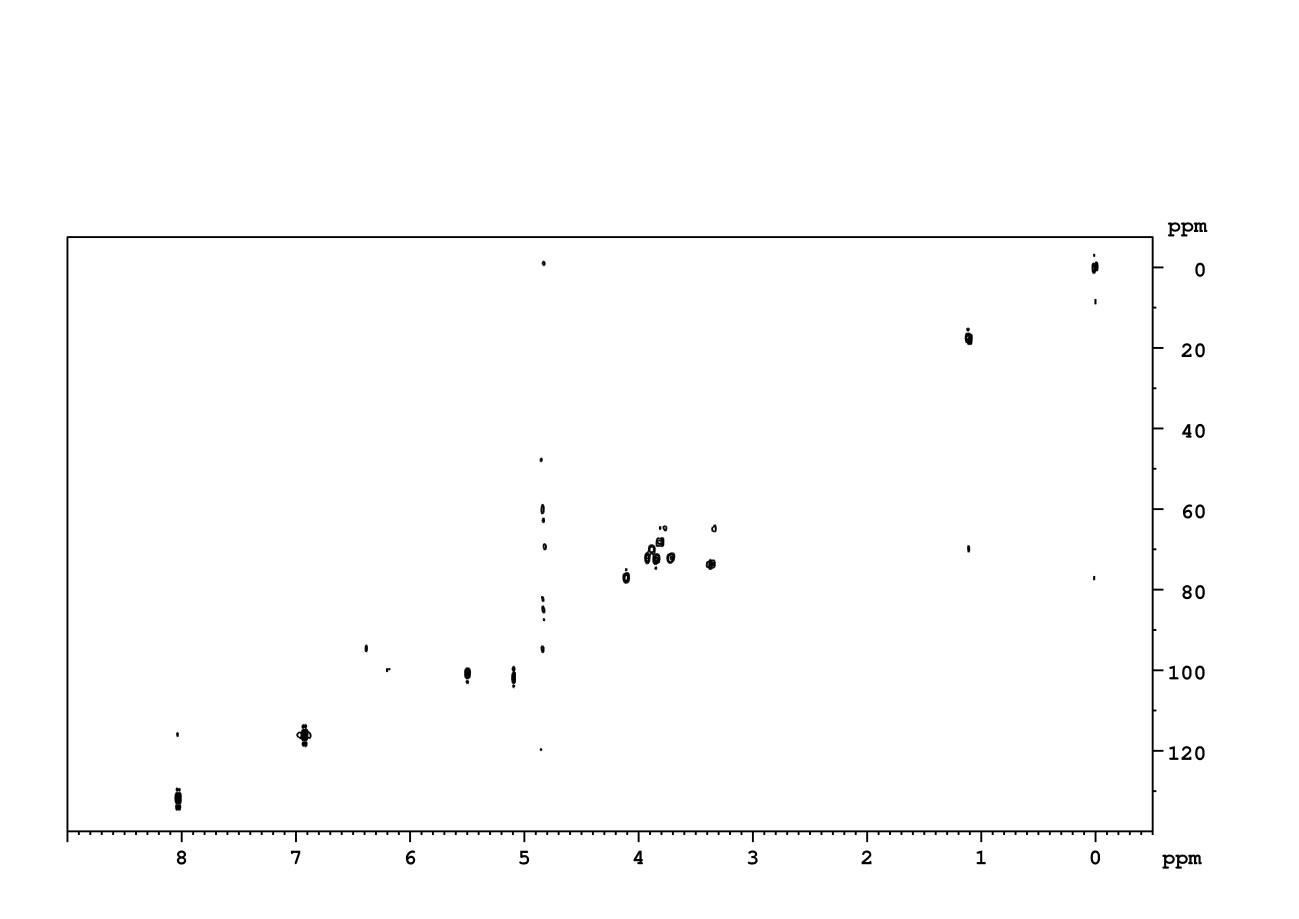


**Figure S7** – Expansion of one-bond ^1^H-^13^C correlation map from HSQC NMR experiment at 600.13 and 150.9 MHz, highlighting correlations for hydrogens of compound 1 (MeOD/TMS, 30 °C).


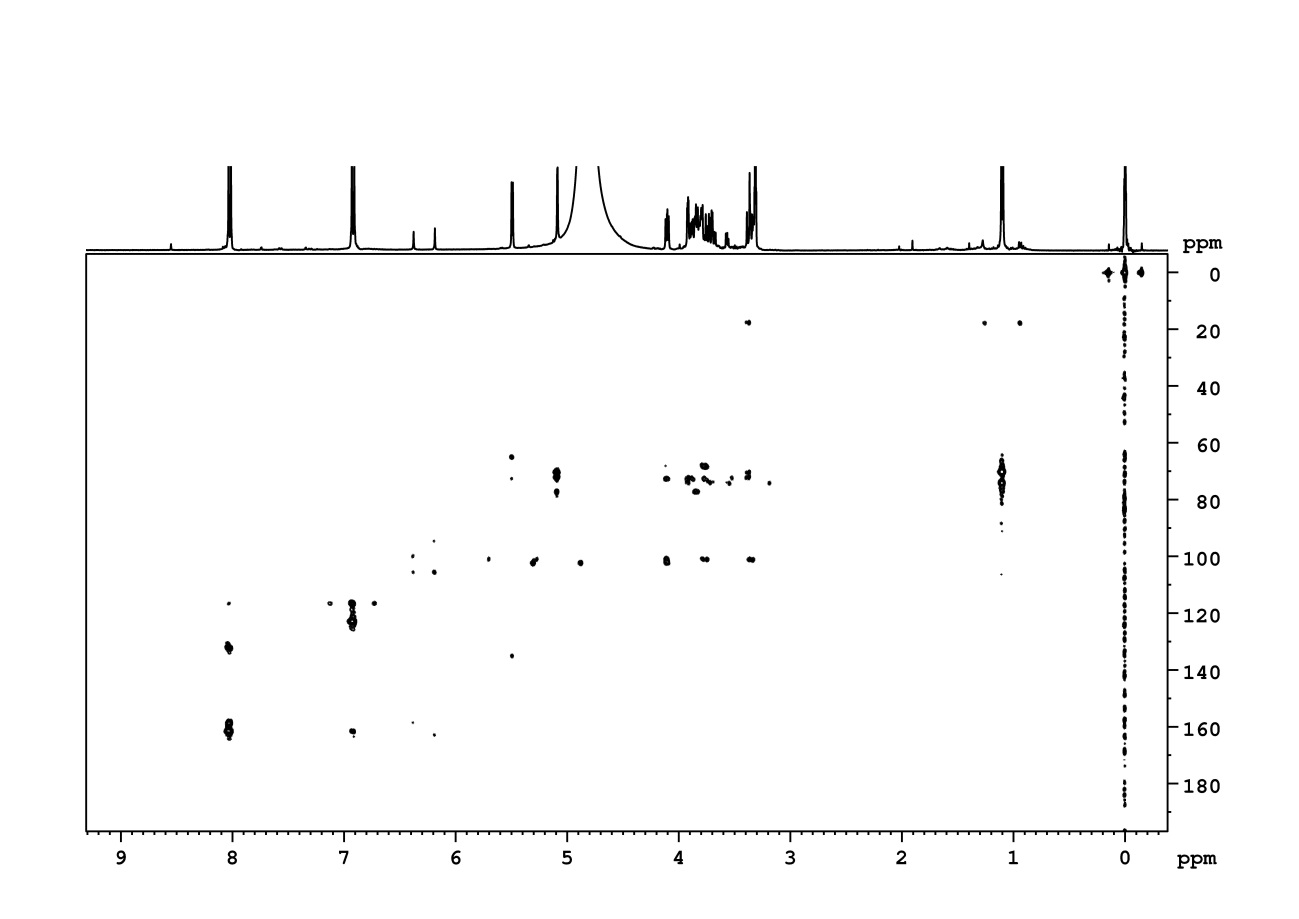


**Figure S8** – Expansion of one-bond ^1^H-^13^C correlation map from HMBC NMR experiment at 600.13 and 150.9 MHz, highlighting correlations for hydrogens of compound 1 (MeOD/TMS, 30 °C).


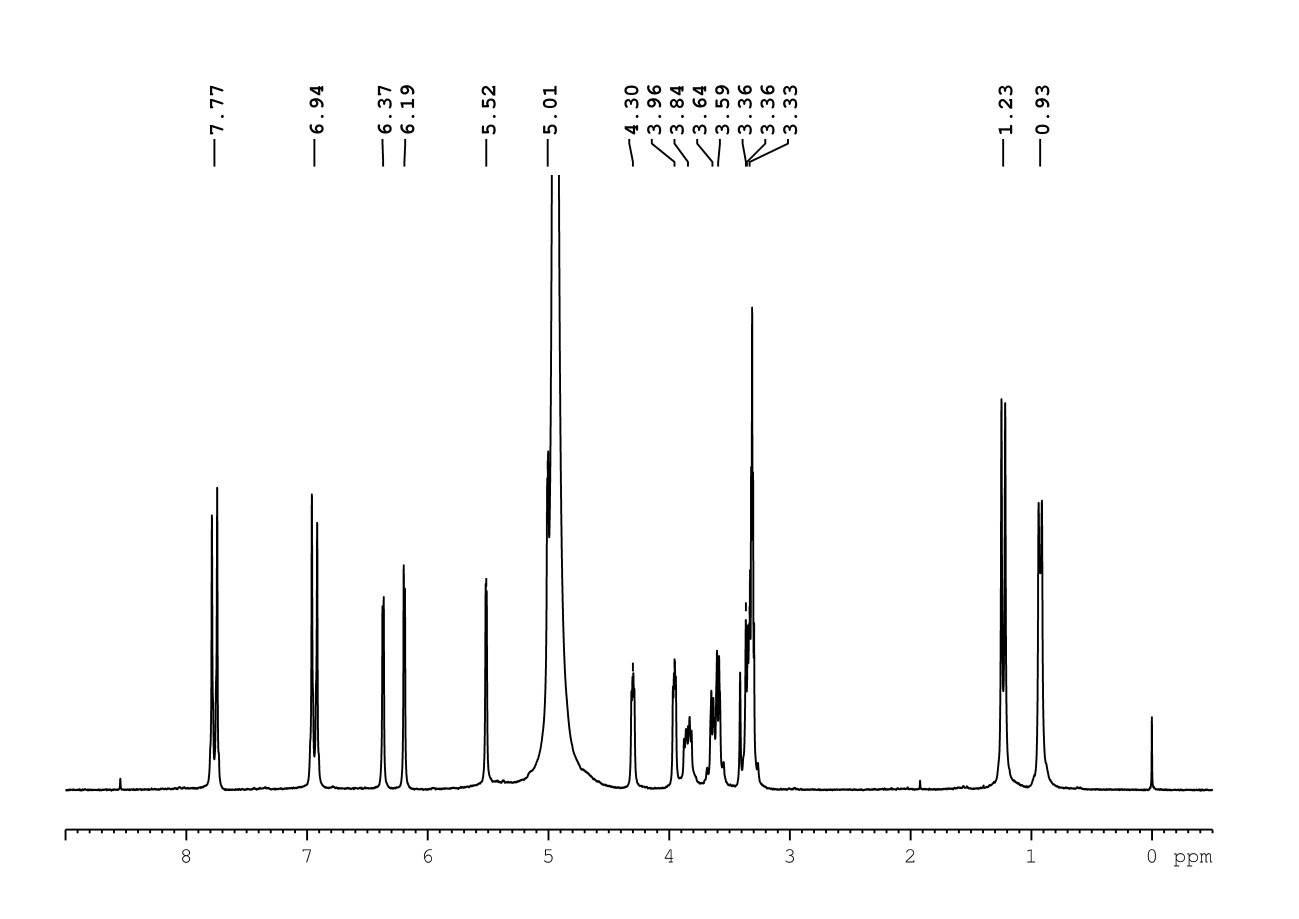


**Figure S9** – Full ^1^H NMR spectrum of compound 2 (MeOD/TMS, 200.13 MHz, 30 °C).


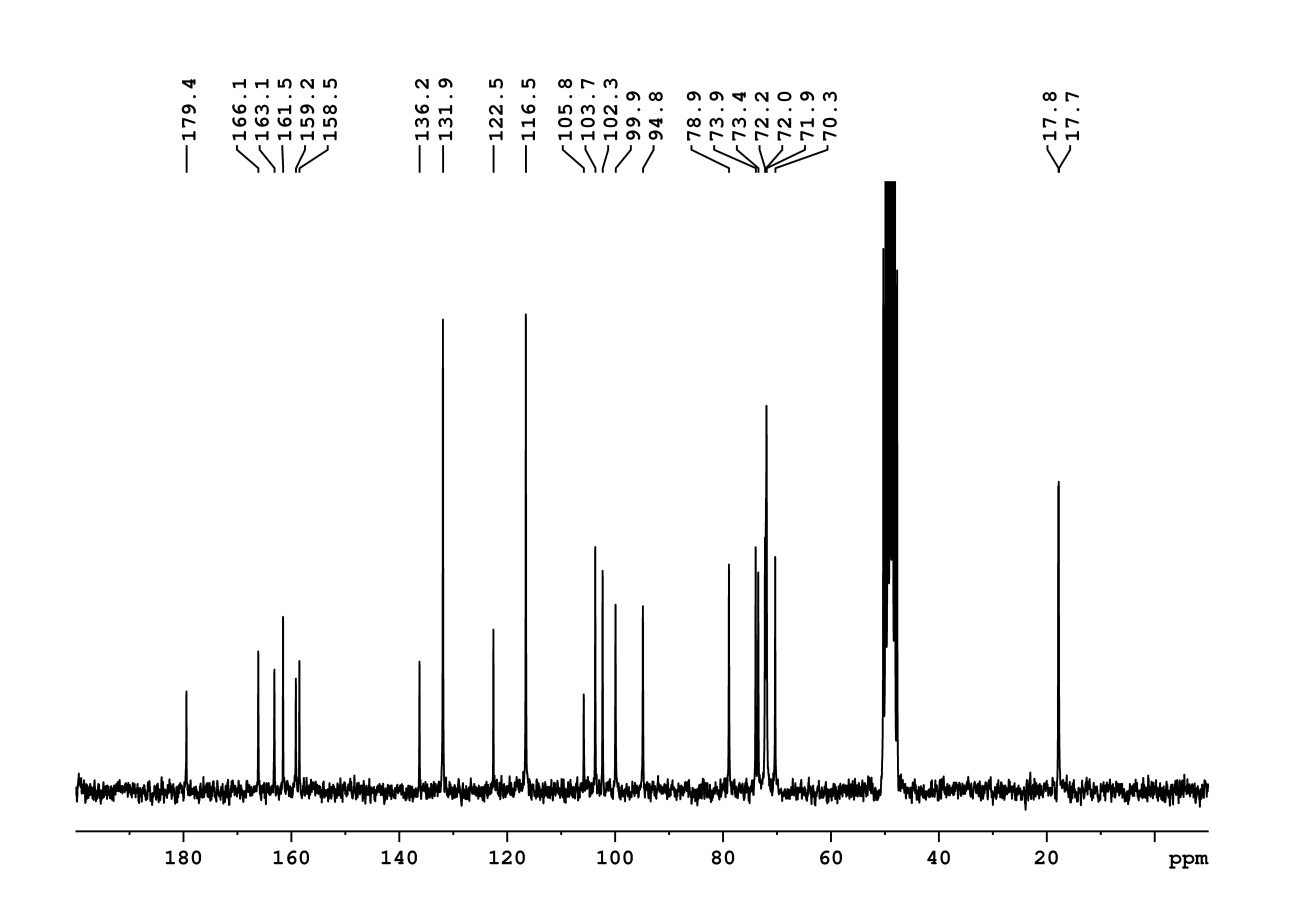


**Figure S10**– Full ^13^C NMR spectrum of compound 2 (MeOD/TMS, 200.13 MHz, 30 °C).

**2. Chromatographic data**


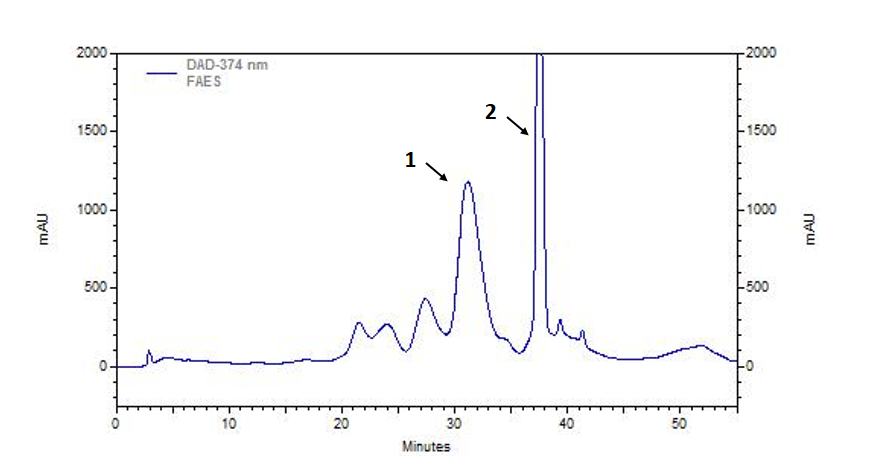


**Figure S11** – Chromatogram of preparative high performance liquid chromatography separation of compounds **1** and **2**, witch correspond to the signals at 31.5 and 37.5 minutes respectively.

**3. Thiobarbituric Reactive Substances**


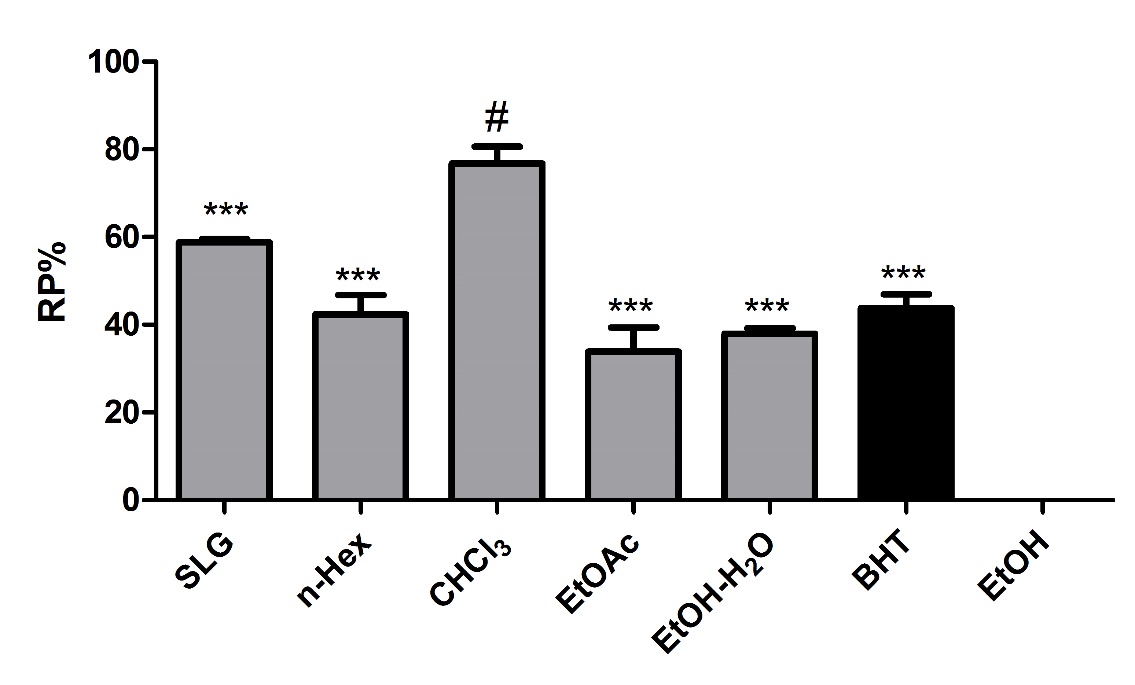


**Figure S12** – Percent of reduction of peroxidation (RP%). Crude ethanolic extract of *Smilax larvata* (SLG), *n*-hexane extract (n-hex), chloroform extract (CHCl_3_), ethyl acetate extract (EtOAc), hydroalcoholic extract (EtOH-H_2_O). Data are expressed as the mean ± S.E.M. ****p*<0.001 when compared to control without antioxidant, #*p*<0.1 when compared to BHT.
